# No effect of odor-induced memory reactivation during REM sleep on declarative memory stability

**DOI:** 10.3389/fnsys.2014.00157

**Published:** 2014-09-01

**Authors:** Maren J. Cordi, Susanne Diekelmann, Jan Born, Björn Rasch

**Affiliations:** ^1^Division of Biopsychology, Institute of Psychology, University of ZurichZurich, Switzerland; ^2^Department of Medical Psychology, Institute of Medical Psychology and Behavioral Neurobiology, University of TübingenTübingen, Germany; ^3^Psychiatric University Hospital, Zurich Center for Interdisciplinary Sleep Research (ZiS), University of ZurichZurich, Switzerland; ^4^Division of Cognitive Biopsychology and Methods, Department of Psychology, University of FribourgFribourg, Switzerland

**Keywords:** rapid eye movement sleep, reactivation, memory stability, hippocampus, declarative object location task

## Abstract

Memory reactivations in hippocampal brain areas are critically involved in memory consolidation processes during sleep. In particular, specific firing patterns of hippocampal place cells observed during learning are replayed during subsequent sleep and rest in rodents. In humans, experimentally inducing hippocampal memory reactivations during slow-wave sleep (but not during wakefulness) benefits consolidation and immediately stabilizes declarative memories against future interference. Importantly, spontaneous hippocampal replay activity can also be observed during rapid eye movement (REM) sleep and some authors have suggested that replay during REM sleep is related to processes of memory consolidation. However, the functional role of reactivations during REM sleep for memory stability is still unclear. Here, we reactivated memories during REM sleep and examined its consequences for the stability of declarative memories. After 3 h of early, slow-wave sleep (SWS) rich sleep, 16 healthy young adults learned a 2-D object location task in the presence of a contextual odor. During subsequent REM sleep, participants were either re-exposed to the odor or to an odorless vehicle, in a counterbalanced within subject design. Reactivation was followed by an interference learning task to probe memory stability after awakening. We show that odor-induced memory reactivation during REM sleep does not stabilize memories against future interference. We propose that the beneficial effect of reactivation during sleep on memory stability might be critically linked to processes characterizing SWS including, e.g., slow oscillatory activity, sleep spindles, or low cholinergic tone, which are required for a successful redistribution of memories from medial temporal lobe regions to neocortical long-term stores.

## Introduction

The fate of a memory after its reactivation strongly depends on the state of the brain. In the brain state of slow-wave sleep (SWS), memories are spontaneously reactivated, and several studies have successfully shown that inducing reactivations during SWS by a reminder activates hippocampal brain areas and improves later memory recall using odors or sounds (Rasch et al., 2007; Rudoy et al., 2009; Diekelmann et al., 2011; Antony et al., 2012; Oudiette and Paller, 2013; Rihm et al., 2014). In contrast, inducing reactivations in a waking brain state by a reminder or active retrieval attempts can lead to a modulation or even forgetting of memories when memory stability is challenged by interfering agents (Nader et al., 2000; Walker et al., 2003), requiring a period of reconsolidation of the memory in order to persist (Nader and Hardt, 2009).

It is assumed that the memory-strengthening effect of hippocampal reactivations during SWS is supported by redistribution of memory representations from hippocampal to neocortical networks in close coordination with SWS specific hippocampal sharp-wave ripples, sleep spindles and slow oscillations (Rasch and Born, 2013). Thereby, hippocampal dynamics and hippocampal-neocortical feedback loops seem to critically depend on a low level of the neurotransmitter acetylcholine, which is characteristic for SWS. Reactivations in this milieu face beneficial conditions for initiating plastic changes in neocortical brain areas (Gais and Born, 2004; Hasselmo and Giocomo, 2006; Rasch et al., 2006). In contrast, levels of acetylcholine are elevated during wakefulness, which may hinder successful consolidation and redistribution of reactivated memories (Hasselmo and Giocomo, 2006). Furthermore, prefrontal process of retrieval monitoring specific for wakefulness might render memories susceptible to interference.

Spontaneous reactivations do not only appear during SWS or wakefulness, but also in the brain state of rapid eye movement (REM) sleep. Neuronal firing patterns in the hippocampus having been active during learning were also active during REM sleep (e.g., Louie and Wilson, 2001). As REM sleep shares several features with waking, including a waking like brain activity and a high cholinergic tone, spontaneous memory reactivations during REM sleep might not be beneficial for memory consolidation. However, several authors have implicated REM sleep and memory reactivations during REM sleep in processes of memory consolidation: first, there is quite consistent evidence from animal studies that REM sleep plays a role in memory consolidation (for a review see Fishbein and Gutwein, 1977). Second, inducing reactivations during REM sleep in animals using fear conditioning procedures in fact improved consolidation of fear (see Hennevin et al., 2007, for a review). Thus, it was suggested that the hippocampal reactivations reflect or contribute to memory processing during REM sleep (Hennevin et al., 1995). However, in contrast to waking and SWS, the consequences of reactivation during REM sleep on memory stability in humans are still unclear.

Here we specifically tested the effect of reactivating hippocampus-dependent, declarative memories during REM sleep on later memory stability including an interference learning task after reactivation. Importantly, we focused on late, REM sleep rich sleep to exclude confounding effects of prior SWS on memory stability. In contrast to the positive effects of cueing during REM sleep in animals, we predicted that inducing reactivations during REM sleep does not stabilizes memories against future interference, because important features critical for a beneficial effect of reactivation on memory consolidation (i.e., hippocampal sharp-wave ripples, slow oscillations, sleep spindles, and a low cholinergic tone) are lacking during REM sleep.

## Materials and methods

### Subjects

Sixteen healthy, nonsmoking young adults, aged 20–34 (23.9 ± 4.02 years, 10 females) were tested in a counterbalanced within subject design. None of the participants reported any irregular sleep-wake cycles, shift working, neurological, psychiatric or endocrine disorders or took any sleep modulating medication. Subjects reported normal sleep (Pittsburgh Sleep Quality Index PSQI < 6) and had neither a nasal infection nor ingested any caffeine or alcohol on the experimental day. They were asked to get up between 7:00 and 8:00 a.m. on experimental days. Subjective reports indicated similar bedtimes (i.e., close to midnight) two days before the experiment for the two experimental conditions, suggesting that participants were in the same circadian rhythm in both conditions. All subjects spent an adaptation night in the sleep laboratory including placement of the nasal mask and electrodes as during the experimental nights. Two subjects were excluded from analyses due to chance level performance at interference learning, and one subject due to learning performance diverging by more than 2 SD from group mean. Subjects gave written informed consent to their participation and were paid 200 Swiss francs. The ethics committee of the University of Zurich approved the study.

### Design and procedure

**Figure 1:**
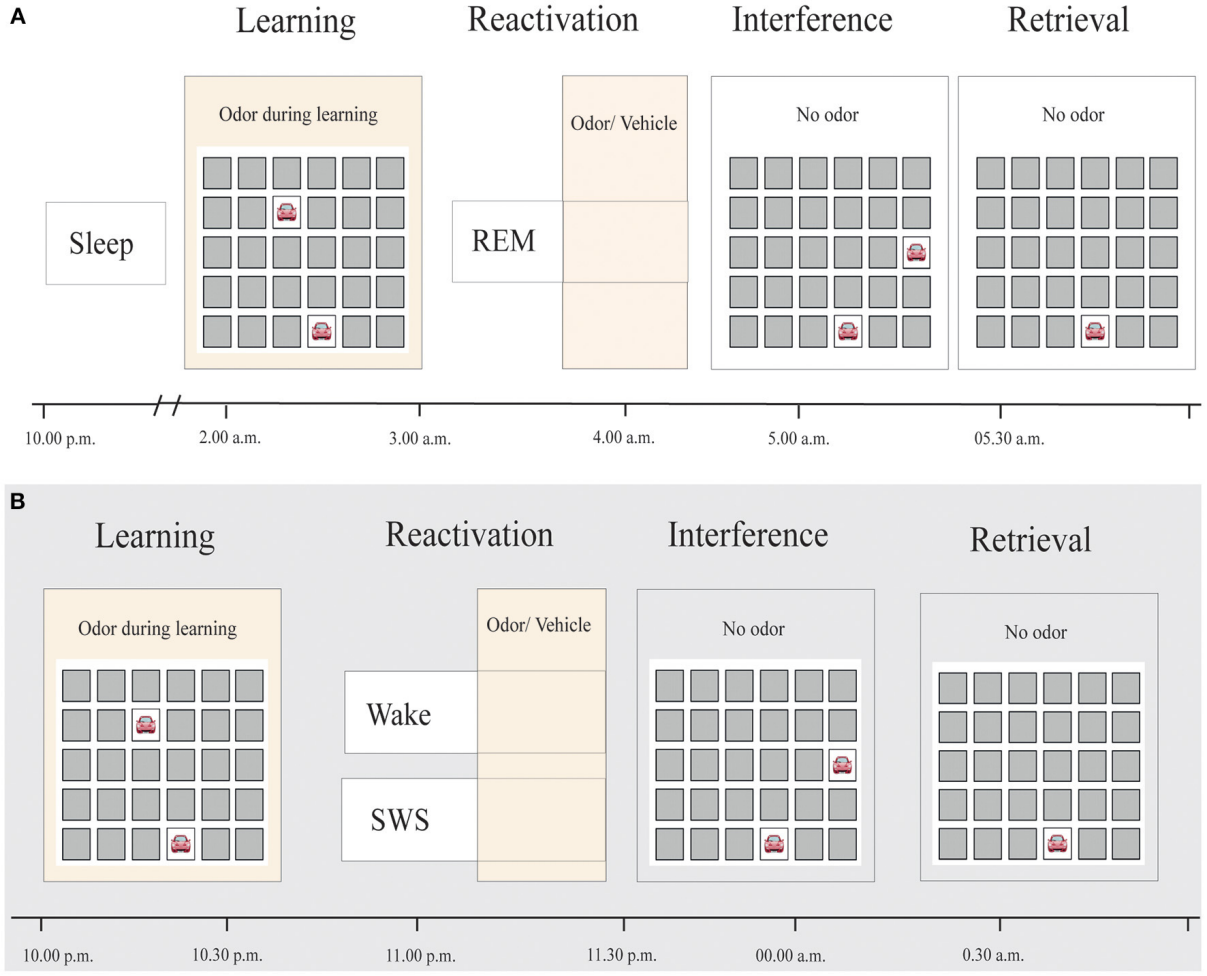
**Experimental procedure. (A)** Subjects slept for approximately 3 h before learning a 2-D object-location task while being exposed to an odor. During subsequent REM sleep, either the same odor or an odorless vehicle was presented for at least 20 min, in a counterbalanced order. After awakening, subjects learned an interfering 2-D object-location task without odor presentation. Retrieval of the original task was tested thereafter. **(B)** Reactivation in Diekelmann et al.'s study (2011), in contrast, occurred either during SWS or wakefulness.

Subjects spent one adaptation night and two experimental sessions in the sleep laboratory. They were fully informed about the session flow. The experimental sessions were separated by at least 7 days. The sessions started at 9:00 p.m. with the attachment of electrodes for electroencephalographic (EEG), electromyographic (EMG) and electrooculographic (EOG) recordings. After filling out standard questionnaires and performing a reaction time test, subjects were allowed to sleep for at least 3 h from 10:30 p.m. on (see Figure 1). Fifteen minutes after awakening, at about 2:00 a.m., participants first performed a reaction time task and an odor detection test to ensure functionality of the olfactometer. Participants then learned the 2-D object-location task in the presence of the odor before they performed the odor detection test and the reaction time task again. Thereafter, participants returned to bed and olfactory stimulation (using a repeated 30-s on/30-s off pattern) was started as soon as polysomnographic recordings indicated stable REM sleep. We stimulated during tonic and phasic REM sleep phases. On one night, participants were re-exposed to the same odor that had been present during prior learning to induce memory reactivation. On the other night, an odorless vehicle stimulus was applied, in a counterbalanced order. Neither the subject nor the experimenter knew about the order of the stimulation. Stimulation was stopped as soon as arousals, awakenings or shifts into other sleep stages were detected. On average, stimulation duration during sleep was 29.77 ± 1.86 min (range 18–45.5 min) following Diekelmann et al.'s (2011) protocol. Post-experimental offline scoring confirmed that 92.55 ± 1.89% of odor stimulation and 92.29 ± 2.98% of placebo stimulation actually took place during REM sleep. Participants were awakened directly after the last reactivation in the REM sleep phase. Shortly after awakening, participants learned the interference 2-D object-location task. After a break of 20 min, recall of the card-pair locations of the original task, learned before sleep, was tested. Participants were asked to perform as well as possible in each of the memory tasks.

### Odor delivery and substance

Odor and placebo were delivered by a computer-controlled olfactometer as described previously (see Rasch et al., 2007, for details). The olfactometer was placed outside the sleep cabins and was connected to the nasal masks via Teflon tubes, such that the subject was not disturbed by any noise accompanying the stimulation procedure. The odor was isobutyraldehyde, diluted in 1,2-propanediol at a concentration of 1:200 (as used in Diekelmann et al., 2011). Odorless propanediol alone served as placebo.

### 2-D object-location task

The two-dimensional object-location memory task, resembling the game “concentration”, was used as described previously (see Rasch et al., 2007; Diekelmann et al., 2011, for details). Learning consisted of remembering the location of 15 card pairs displaying animals and everyday objects on the computer screen. Card pairs were uncovered sequentially during learning phase until all pairs had been shown twice. Thereafter, the first card of each card pair was uncovered and subjects were required to indicate the correct position of the second card by a mouse click on the respective field. Feedback was provided indicating the correct location of the second card. This cued recall procedure was repeated until at least 60% of responses were correct. The odor presentation during learning was event-locked, being initiated with onset of the presentation of the first card and enduring until the presentation of both cards was discontinued. The final recall of this task after sleep was similar to the cued recall procedure during learning except that no odor was presented and that only one cued recall trial was administered. As dependent variable (“memory retention across sleep”), the relative amount of correctly retrieved card pair locations was used, with performance at learning set to 100%.

Interference learning was conducted with the same task, including the same card pairs, but the position of the second card of each pair was changed. No odor was presented during interference learning and there was only one single cued recall trial following learning. For the two experimental sessions, two parallel versions of both, the object-location task as well as its interference equivalent, were used. Parallel versions included different pictures.

### Vigilance and subjective sleepiness

Reaction times were assessed in a reaction time task before the first sleep episode, before and after learning as well as after recall of the original 2-D object location task to assess general alertness. A red dot randomly appeared on a screen and subjects had to press the space key as soon as they recognized the dot. 45 trials were included. Subjective sleepiness was assessed using the Stanford Sleepiness Scale before the first sleep period and at the end of the experimental session.

### Cortisol measures

Cortisol was measured with saliva tests (Sarstedt, Germany). Saliva cortisol concentrations were measured using a commercially available luminescence immune-assay (IBL, Hamburg, Germany) with intra- and interassay coefficients of variation <5%. Cortisol measures were taken before and after the first and second night half and after recall of the original 2-D object location task at the end of each experimental session.

### Polysomnographic recordings

EEG was recorded from three scalp electrodes (Fz, Cz, and Pz according to the international 10–20 system) and an averaged mastoid reference. Data was prepared using the VisionAnalyzer 2.0 (Brain Products, Germany) and filtered according to the settings suggested by the American Academy of Sleep Medicine (AASM). Additionally to the online scoring of sleep stages, sleep was scored offline using 30 s periods according to standard criteria (Iber et al., 2007) as wake, sleep stages N1–N3 and REM sleep, by two sleep experts.

For a more fine-grained analysis of sleep during the second night half power spectral analyses were run on EEG recordings during Non-REM and REM sleep. Data of 30 s of sleep were segmented into artifact-free blocks of 4096 data points (≈ 8.2 s) with an overlap of 409 data points, respectively to achieve a resolution of 0.2 Hz. Before calculating power using fast Fourier transform, a Hamming Window of 10 % was applied on the data points. Individual area (μV^*^ ms) information was determined for slow wave activity (SWA) during Non-REM (1–4.5 Hz), theta during REM sleep (4.5–8 Hz), and slow (11–13 Hz) and fast spindles (13–15 Hz) during Non-REM sleep.

### REM analysis

REM density was calculated by dividing the number of 1-s periods during REM sleep that contained REMs by the total number of 1-s REM sleep epochs (Ficca et al., 2004). Rapid eye movements were detected automatically as rapid changes in the EOG signal (> 0.8 mV/s) after movement artifact rejection.

### Statistical analyses

Data was analyzed using paired *t*-tests including the factor “Reactivation” (odor vs. placebo). For the critical investigation of the differential influence of brain state on memory stability, a comparison of data with the previous study (Diekelmann et al., 2011) was conducted with a repeated measures ANOVA with the within-subjects factor “Reactivation” (odor vs. placebo) and the between-subjects factor “Study” (SWS vs. wake vs. REM) on recall performance. The level of significance was set to *p* = 0.05. Greenhouse Geisser corrections were used whenever indicated by significant tests of sphericity. For descriptive values, mean ± s.e.m. are indicated.

## Results

### Effect of induced memory reactivation during REM sleep on memory stability

**Figure 2:**
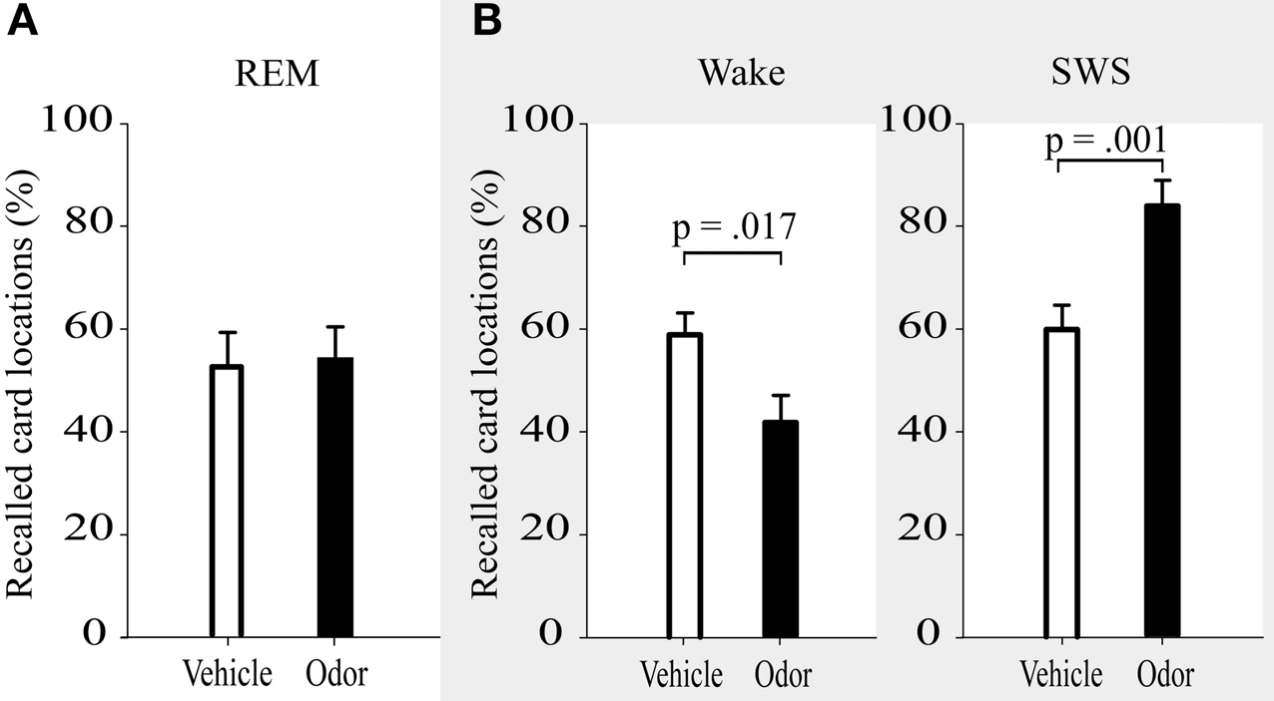
**Recall of card locations (%) was not differentially affected by interference learning after reactivation in REM sleep (A), but showed impairments after reactivation during wakefulness and enhanced resistance toward interference after reactivation in SWS (B, data adapted from Diekelmann et al., 2011).** Values are means ± s.e.m.

In contrast to the hypothesis of the beneficial role of memory reactivations during REM sleep, inducing reactivations during REM sleep had no influence on memory stability. After odor-induced memory reactivation during REM sleep, participants remembered 54.16 ± 5.93% of the learned locations, whereas they correctly recalled 52.86 ± 6.49% after presentation of the odorless vehicle stimulus (*p* = 0.87, Figure 2A and Table 1). Learning performance (number of recalled card pairs at the end of learning) did not differ significantly between the two conditions (9.92 ± 0.26 vs. 10.69 ± 0.37, *p* = 0.13) and learning of the interference task was also highly comparable (6.07 ± 0.83 vs. 5.67 ± 0.82, *p* = 0.72).

**Table 1:** **Performance on the 2-D object location task**.

	**Odor**	**Placebo**	***P***
Learning	9.92 ± 0.26	10.69 ± 0.37	0.13
Number of trials	3.39 ± 0.58	2.54 ± 0.45	0.10
Absolute change	−4.62 ± 0.66	−5.00 ± 0.69	0.68
Relative change	54.16 ± 5.93	52.86 ± 6.49	0.87
Interference learning	6.07 ± 0.83	5.67 ± 0.82	0.72

Absolute recall performance during learning of the original object-location task, number of trials needed to reach criterion, absolute and relative change from learning to retrieval, and absolute recall performance during learning of the interference task. Mean ± s.e.m. are indicated.

In addition, we directly compared the effects of REM sleep reactivation on memory to the results of the SWS reactivation condition and waking reactivation condition from our previous study (Diekelmann et al., 2011). The learning and odor-induced reactivation protocol was identical in both studies (Figure 1B). However, time of learning was 10:00 p.m., and retrieval took place at around 0:30 a.m. in the previous study, thus examining a period of early SWS rich sleep vs. a corresponding waking interval, in contrast to the late REM sleep rich interval in the present study. Descriptively, the odor reactivation-induced influence on memory stability (in comparison with placebo) was +23.38 ± 5.48% for reactivation during SWS, 1.30 ± 8.01% for reactivation during REM sleep and −17.96 ± 6.37% for reactivation during waking [*F*_(2, 34)_ = 9.03, *p* = 0.001, η^2^ = 0.35]. This expressed itself in a significant interaction when comparing the REM sleep reactivation with the SWS reactivation group, which indicated a robust stabilizing effect of memory reactivation during SWS that was not evident after reactivation during REM sleep [*F*_(1, 23)_ = 5.00, *p* = 0.035, η^2^ = 0.18; Figure 2). There was also a statistical trend for the interaction between reactivation during REM sleep as compared to waking, with a destabilization of memories after reactivation during wakefulness but not during REM sleep [*F*_(1, 23)_ = 3.47, *p* = 0.08, η^2^ = 0.13]. Generally, SWS as well as waking groups outperformed participants in the REM sleep group in recall of card pairs independent of reactivation or placebo conditions [main effect condition *F*_(2, 34)_ = 9.10, *p* = 0.001, η^2^ = 0.35].

### Control variables

There were no differences in encoding of the original memory task between the studies [*F*_(2, 34)_ = 0.43, *p* = 0.66 for number of remembered pairs, *F*_(2, 34)_ = 0.56, *p* = 0.58 for number of trials]. Furthermore, learning of the interference task also did not differ significantly between the REM sleep group (5.89 ± 0.62) and the SWS group (5.63 ± 0.65), *t*_(23)_ = −0.31, *p* = 0.76. As reported previously, interference learning in the wake group was better than in the sleep groups [9.13 ± 0.65, *F*_(2, 34)_ = 9.10, *p* = 0.001], which was already ruled out as confounding factor by a subgroup analysis with matched interference learning performance (Diekelmann et al., 2011). We observed no difference in sleep stage distribution between odor and placebo condition, neither during late REM sleep rich sleep during which olfactory stimulation was applied (all *p* ≥ 0.14, see Table 2 for descriptive values) nor during early SWS rich sleep before learning (all *p* ≥ 0.23). Furthermore, we did not find any significant differences between reactivation and placebo nights in oscillatory power for the theta band during REM sleep (*p* ≥ 0.31) or for the SWA (*p* ≥ 0.23), slow spindle (*p* ≥ 0.11) and fast spindle bands (*p* ≥ 0.15) during Non-REM sleep in none of the electrodes (i.e., Fz, Cz, Pz). In addition, there were no significant differences between odor and placebo conditions in REM density (*p* = 0.18), 1 s segments in which eye movements occurred (*p* = 0.64), subjective sleepiness ratings (*p* ≥ 0.78), reaction times during learning and retrieval (all *p* ≥ 0.24), cortisol levels (*p* ≥ 0.08), or odor detection before and after learning (*p* ≥ 0.17) (see Table 3). Finally, memory retention differences between both nights did neither correlate with the difference in sleep stages (*p* ≥ 0.08) nor the difference in each of the control variables (*p* ≥ 0.23). Further, neither REM density (*p* = 0.76) nor number of segments in which eye movements occurred (*p* ≥ 0.24) during odor presentation correlated with the recall memory performance of the original task after sleep.

**Table 2:** **Sleep stages for the early night (before learning) and late night (after learning with odor/placebo stimulation)**.

**Sleep stages (in minutes)**	**Early night**			**Late night**		
	**Odor**	**Placebo**	***p***	**Odor**	**Placebo**	***p***
Wake	2.41 ± 1.53	8.69 ± 4.74	0.24	4.27 ± 2.24	4.46 ± 1.95	0.94
N1	8.89 ± 1.13	9.50 ± 1.95	0.73	11.00 ± 2.20	9.77 ± 1.95	0.54
N2	71.46 ± 5.87	66.54 ± 7.13	0.49	65.12 ± 8.67	62.19 ± 5.43	0.76
N3	89.31 ± 5.62	89.89 ± 10.63	0.96	20.69 ± 4.16	32.156 ± 6.52	0.14
REM	24.89 ± 3.18	20.00 ± 2.79	0.23	35.62 ± 3.02	36.23 ± 4.11	0.88
Sleep latency	20.96 ± 6.80	16.96 ± 3.84	0.44	19.35 ± 2.42	23.31 ± 4.55	0.42
SWS latency	14.27 ± 1.60	18.73 ± 5.77	0.40	40.85 ± 9.75	39.35 ± 11.47	0.90
REM latency	106.31 ± 11.76	117.00 ± 11.76	0.46	64.04 ± 5.22	57.65 ± 4.52	0.26

Mean ± s.e.m. are indicated.

**Table 3:** **Values of the control variables before and after early and late sleep**.

	**Before**			**After**		
	**Odor**	**Placebo**	***p***	**Odor**	**Placebo**	***p***
**EARLY SLEEP**						
Objective vigilance (RT)	273.53 ± 10.34	267.90 ± 9.76	0.55	282.86 ± 15.30	271.92 ± 12.42	0.42
Cortisol level	2.75 ± 0.57	2.72 ± 0.36	0.97	3.24 ± 0.50	2.06 ± 0.36	0.08
**LATE SLEEP**						
Objective vigilance (RT)	279.50 ± 13.45	275.57 ± 11.51	0.59	281.45 ± 13.42	276.02 ± 12.03	0.24
Cortisol level	7.04 ± 1.54	6.45 ± 1.13	0.67	13.65 ± 2.60	10.59 ± 2.26	0.38
Subjective sleepiness	3.08 ± 0.18	3.15 ± 0.19	0.78	3.31 ± 0.29	3.31 ± 0.35	>0.99
Odor detection level	8.92 ± 0.21	9.39 ± 0.27	0.17	8.77 ± 0.32	8.85 ± 0.34	0.82

Mean ± s.e.m. are indicated. Values for objective vigilance and cortisol measures are indicated for the measures before the first and second sleep period. Subjective sleepiness was measured before the first sleep period and in the morning after the second sleep period. Odor detection was measured before and after the second sleep period in which reactivation took place.

## Discussion

In contrast to several studies suggesting a functional role of reactivations during REM sleep for memory consolidation, inducing reactivations of hippocampus-dependent, declarative memories during REM sleep by memory-associated odors did not improve later memory resistance. In this respect, our data is in line with previous studies attributing no specific role of REM sleep in sleep-dependent processes of declarative memory consolidation (Rasch et al., 2007). Several studies using night-half paradigms have consistently shown that declarative memories benefit from early, SWS rich sleep, but not from late, REM rich sleep (Plihal and Born, 1997). Furthermore, selective REM sleep deprivation typically does not impair declarative memory consolidation (Chernik, 1972; Lewin and Glaubman, 1975). However, some reports of hippocampal memory reactivation during REM sleep exist (Poe et al., 2000; Louie and Wilson, 2001), and REM-associated dreaming activity has been long suspected to play a role in processes of memory consolidation (Crick and Mitchison, 1995). Despite the possible existence of spontaneous memory reactivation during REM sleep, here we show that experimentally inducing reactivation does not stabilize declarative memories. Importantly, we used an established paradigm which resulted in improved memory when the odors were applied during SWS as now shown in three independent studies (Rasch et al., 2007; Diekelmann et al., 2011; Rihm et al., 2014). Enhanced memory performance (Rasch et al., 2007) and stability (Diekelmann et al., 2011) after induced memory reactivation with the learning-associated odor during SWS were accompanied by hippocampal brain activations. According to the active system consolidation account, sleep-dependent memory consolidation depends on a close interaction between hippocampal memory reactivation, slow oscillations, and sleep spindles resulting in a strengthening and reorganization of memories during sleep (Rasch and Born, 2013). Besides the lack of slow oscillations and sleep spindles during REM sleep, the high acetylcholine level during REM might explain why the induction of reactivation during this sleep stage does not stabilize memories. During SWS, hippocampal reactivations are assumed to feed back to neocortical areas to initiate plastic changes in neocortical brain areas involved in long-term memory storage. It is assumed that the disinhibition of these feedback projections critically depends on low levels of the neurotransmitter acetylcholine, prevalent during SWS (Hasselmo and Giocomo, 2006; Rasch et al., 2006). This is supported by studies showing that the increase of acetylcholine during SWS completely blocks declarative memory consolidation (Gais and Born, 2004). Thus, this transmitter milieu of low cholinergic tone seems to be a prerequisite for the beneficial role of spontaneous and induced reactivations on memory stability. Future studies still need to test, which of these parameters are responsible for the lack of the beneficial effect of reactivations during REM sleep for memory stability.

Interestingly, although REM sleep shares several features with the wake state, inducing reactivation during REM sleep did also not destabilize memories as observed in reconsolidation studies during waking (Nader and Hardt, 2009). It can be speculated that waking consciousness, awareness, and a functional “encoding mode” might be important factors contributing to a destabilization of memories after reactivation. For example in rats, inhibition of protein synthesis during wakefulness blocked memory reconsolidation only when new encoding of information was involved during the time of reactivation (Morris et al., 2006). This encoding mode might be reflected in the prefrontal activations following memory reactivation during wakefulness as was shown in Diekelmann et al. (2011). Thus, reactivation might only induce a destabilization of memories when conscious encoding of new information occurs simultaneously, possibly even merely in case of a “conflict” between new and reactivated information and a need for an “updating” of the reactivated information with respect to the new one. In contrast, no new or conflicting information is simultaneously encoded during REM sleep, which might explain the lack of reactivation-induced destabilization during this sleep stage.

It might be argued that odors are not capable of reactivating memories during REM sleep. We consider this explanation unlikely, because odors administered during REM sleep are readily processed, can influence dreams (Trotter et al., 1988) and can be conditioned to preceding tones (Arzi et al., 2012). In addition, previous studies have shown that memories can be reactivated during REM sleep using fear-conditioned tones in animals (see Hennevin et al., 2007, for a review). Importantly, several studies implicate REM sleep in procedural and emotional learning processes (Nishida et al., 2009, for reviews see Smith, 2001; Rasch and Born, 2013), and it might be possible that reactivation of emotional memories or procedural memories during REM sleep (instead of neutral declarative memories) benefits their consolidation.

Of particular note is that we specifically tested effects on memory stability after REM sleep reactivations. Thus, our data does not exclude other memory functions of reactivations during REM sleep. For instance, Sterpenich et al. (2014) similarly showed no effect of reactivations during REM sleep on general recognition performance. However, reactivating memories during REM sleep increased both hit rates as well as false alarm rates and changed the associated neural activity during recognition testing. The authors concluded that reactivations during REM sleep might modify memory traces resulting in a better integration and establishment of associative connections. Our data does not contradict this interpretation.

Independent of the reactivation effect, general recall performance after sleep was lower in the present compared to our previous study, possibly due to different times of encoding, consolidation and recall of the original object-location task and learning of the interference task (Figure 1). However, despite different times of learning and possible concomitant, confounding circadian influences and prior sleep effects, we did not find performance differences in encoding performance. Moreover, consolidation in the current study occurred during a late REM sleep rich sleep interval, which *per se* is typically not beneficial for declarative memories (Rasch and Born, 2013). Furthermore, elevated levels of cortisol in the morning might have hindered memory recall while supporting learning of the interference memory (Wolf, 2009). Finally, participants slept longer in the current than in the previous study and thus, the length of the retention interval was different (138 vs. 40 min). However, we did not find any significant correlation between sleep duration and recall performance. Importantly, the effect of REM sleep reactivation on memory stability *per se* depends on the comparison within one and the same study (i.e., reactivation vs. placebo), rendering confounding effects of the above-mentioned factors rather unlikely. Further it must be considered that participants still performed at a rather high level, excluding bottom effects or performance at chance level.

In sum, our study provides no evidence for an effect of reactivation during REM sleep on the stabilization of declarative memories. Even though spontaneous memory reactivations might exist during REM sleep, they might have no functional effect on stabilizing processes of declarative memory consolidation during sleep. Our results suggest that this might depend on SWS specific events like slow oscillations or spindles or the low cholinergic tone during SWS. Future studies need to test whether emotional or procedural memories profit from inducing memory reactivations during REM sleep. Furthermore, other qualitative memory changes might have resulted from reactivations, which were not measurable with our design.

## Author contributions

Maren J. Cordi, Susanne Diekelmann, and Björn Rasch designed the experiment, Maren J. Cordi collected the data, Maren J. Cordi, Susanne Diekelmann and Björn Rasch analyzed the data. All authors wrote the manuscript, discussed results and approved the final version.

### Conflict of interest statement

The authors declare that the research was conducted in the absence of any commercial or financial relationships that could be construed as a potential conflict of interest.
